# Electronic nicotine delivery system design and aerosol toxicants: A systematic review

**DOI:** 10.1371/journal.pone.0234189

**Published:** 2020-06-04

**Authors:** Alexandra M. Ward, Rola Yaman, Jon O. Ebbert

**Affiliations:** 1 Inhaled Particle Aerosol Lab, Nicotine Dependence Center, Mayo Clinic, Rochester, Minnesota, United States of America; 2 Queen’s University, Kingston, Ontario, Canada; Battelle Memorial Institute, UNITED STATES

## Abstract

**Background:**

Electronic nicotine delivery systems (ENDS; e-cigarettes), consisting of a battery, heating element and e-liquid, have evolved significantly with wide variation in design, components, operating powers, and chemical constituents. Generated aerosols have been reported to contain potentially toxic substances. We conducted a systematic review to assess what is known about the presence of toxicants in ENDS aerosols in order to inform how system design could mitigate risk.

**Methods:**

Articles reporting on or evaluating design characteristics of ENDS and aerosol constituents were included and summarized.

**Results:**

The search identified 2,305 articles, of which 92 were included after full-text review. Findings were grouped into 6 major categories of potentially harmful chemicals: carbonyls, volatile organic chemicals, trace elements, reactive oxygen species and free radicals, polycyclic aromatic hydrocarbons, and tobacco-specific nitrosamines. In general, higher concentrations of aerosol toxicants are associated with increased power or voltage. Aerosol toxicants are also associated with e-liquid flavoring agents existing as primary ingredients or as products of thermal degradation.

**Conclusions:**

Improved ENDS design can reduce toxicant levels. Additional research is needed to develop a framework for optimizing system characteristics to minimize exposure, especially with respect to heating power and e-liquids. Both manufacturers and regulatory agencies have roles in reducing toxicants and potential health risks from ENDS.

## Introduction

Electronic nicotine delivery systems (ENDS) consist of a battery, a heating element (usually consisting of a coil and wick), and liquid (“e-liquid”). The heated coil aerosolizes the e-liquid which consists of propylene glycol (PG), vegetable glycerin (VG), nicotine, and flavoring agents. ENDS were introduced commercially in China in 2003 and to the United States and Europe between 2006 and 2007 [1]. The ENDS market was a $19.3 billion global industry in 2018 [2] which is expected to increase to $58 billion by 2026 [3]. ENDS have been proposed as a smoking cessation strategy [4], or for smoking reduction [5], but concerns also exist that ENDS may serve as a “gateway” to conventional tobacco products among adolescents [6].

The aerosols produced by ENDS have been reported to contain potentially toxic substances, which the user inhales [7]. Previous literature reviews evaluated aerosol carbonyls [8], trace metals [9], and methodological approaches to analysis [10]. A broader review [11] evaluated carbonyls, volatile organic compounds (VOCs), metals, polycyclic aromatic hydrocarbons (PAHs), and tobacco-specific nitrosamines (TSNAs) in aerosols, as well as flavors and solvents in refill solutions, and cartridges. No reviews have summarized the literature relating to the impact of ENDS design on all identified aerosol toxicants. Potential toxicants originate from e-liquid components, degradation of ENDS materials, and reactions between the device and e-liquid delivering chemicals such as VOCs, trace elements [12], and carbonyls such as formaldehyde, acrolein [13], and acetaldehyde [14]. Investigators have suggested that differences in the engineering process, individual modifications [15], and design features affect aerosol composition [16].

We conducted a systematic review of the literature in order to collate and integrate the literature assessing the design characteristics of ENDS associated with the production or release of potentially harmful substances in inhaled aerosols. We approached our review from the perspective of advancing understanding of how ENDS design could inform scientific and policy discussion around how to modify potential risks associated with their use.

## Methods

A registered protocol does not exist for this systematic review. A PRISMA checklist is available in supporting information (S1 Checklist).

### Inclusion criteria

The review included articles reporting on (i.e., listing ENDS characteristics) or evaluating (i.e., assessing how design differences impact output) ENDS design characteristics and aerosol constituents, with a specific focus on the measurement of chemical species in aerosols produced by heated-coil ENDS.

### Exclusion criteria

We excluded articles first by duplicates (n = 287), then a title and abstract screening, and finally by a full-text screening. Irrelevant studies included (n = 1,835), but were not limited to, articles not assessing ENDS aerosol, letters, commentaries, or other reviews, second-hand or third-hand aerosol/residue assessments from ENDS, and environmental exposure studies. Additionally, studies retrieved that were not available in English or were published anonymously were also eliminated at this stage. At the full-text screening, studies were eliminated upon the basis of 4 criteria: wrong study design (n = 32; e.g., indirect measures of toxicity or cytotoxicity studies, exhaled breath or saliva studies, or only analyzed e-liquid), no primary data (n = 28; e.g., perspective, comments, or responses to previous literature), no full text (n = 17), and assessed major aerosol constituents only (n = 14; studies *exclusively* analyzing the known major and expected aerosol constituents nicotine, PG, and VG) were also excluded as these are expected aerosol constituents when liquids containing them are aerosolized.

We defined risk of bias *a priori* as the utilization of analytical techniques employed by research groups externally (Group A), internally (Group B), or non-replicated (Group C) for a specific major chemical category. Because we did not quantitatively combine study results, no articles were excluded based on risk of bias.

### Search strategy

We searched Ovid MEDLINE, Epub Ahead of Print, In-Process & Other Non-Indexed Citations, Daily and Versions electronic databases, selecting articles published from 1946 until May 07, 2020. We employed the following keywords: "electronic nicotine device," “vape,” “vaping,” "electronic nicotine delivery system," "electronic cigarette," "e-cigarette," “ecig,” and "e-cigs." These terms were combined with the following terms: “acetaldehyde,” “acrolein,” “aerosol,” “air pollutant,” “aldehyde,” "aromatic amine," “atomizer,” “carbonyl,” “coil wire,” “e-liquid,” “exp flavoring agents,” “exp metals, heavy,” “exp temperature,” “formaldehyde,” "free radical," “glycerol,” "heavy metal," “nitrosamine,” “phenol,” "polycyclic aromatic hydrocarbon," “propylene glycol,” “thermal degradation,” “toxic,” “toxicity,” “vocs,” “volatile,” and "volatile organic compounds".

## Results

**Fig 1:**
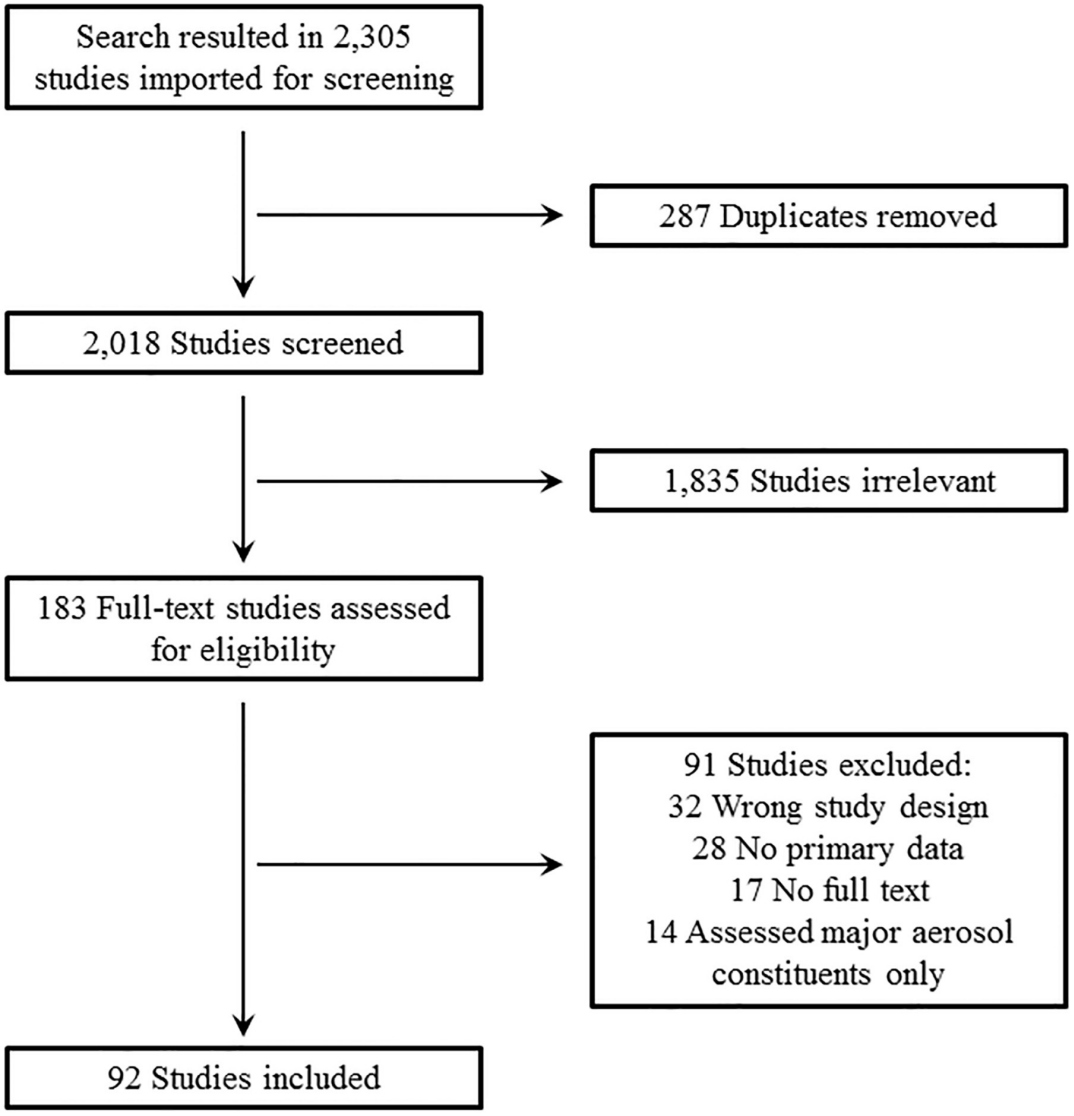
Methodological approach for systematic review and article selection.

Our search strategy identified 2,305 articles, and full-text review resulted in inclusion of 92 studies (Fig 1). We grouped studies into 6 major chemical groups. Five were included on the basis of their designation as “harmful and potentially harmful constituents” in tobacco products by the US Food & Drug Administration (FDA) [17]: carbonyls, VOCs, trace elements, PAHs, and TSNAs. In addition, we added free radicals and reactive oxygen species (ROS) because of their potential to cause tissue injury and disrupt physiological function [18]. If a particular chemical did not fit into one of these categories, it was categorized as “other” and not included in the presented tables.

Because there is overlap in the physical properties of categories, chemicals were sorted on the basis of general consensus in the literature as well as physical characteristics. The carbonyl category consisted of chemicals containing a carbonyl moiety; examples include formaldehyde and acetaldehyde. VOCs were defined as organic compounds with a boiling point between 50 and 250 °C [19], such as benzene and styrene. Elemental analysis was required for inclusion in the trace elements category; common examples include copper (Cu), lead (Pb), and zinc (Zn). ROS and free radicals included studies analyzing ROS, free radicals, or flux in ROS. PAHs included compounds exclusively containing carbon and hydrogen atoms and at least 2 benzene rings bonded in particular arrangements such as clustered, angular, and linear [20]. Common examples seen in the ENDS literature are benzo[a]pyrene and chrysene. TSNAs are N-nitroso derivatives of pyridine alkaloids such as nicotine and nornicotine that are naturally occurring in tobacco and increase throughout the curing process [21]; N’-nitrosonornicotine (NNN) and 4-(methylnitrosamino)-1-(3-pyridyl)-1-butanone (NNK) are notable examples. Articles were not combined quantitatively due to heterogeneity in collection methods, analytical methods, device parameters, and reported units.

Our search identified publications reporting ENDS characteristics and major toxicant chemical groups in aerosols (Table 1; S1 Table). We identified carbonyl compounds as the most commonly analyzed toxicants and further characterized articles by the specific carbonyl chemical analyzed (Table 2). The majority of these studies reported power, device type (i.e., manufacturer or other device identifying characteristics regarding atomizers, cartomizers, or clearomizers), and e-liquid flavor (i.e., commercially available or laboratory-prepared) and evaluated aerosol constituents of formaldehyde, acetaldehyde, and acrolein.

**Table 1:** Number of studies reporting ENDS^a^ characteristics and measured major chemical groups in aerosol.

Characteristic	Carbonyls	VOCs	Trace Elements	ROS and Free Radicals	PAHs	TSNAs
Power (W)	**28** [22–49]	**7** [39, 44, 47–51]	**5** [49, 51–54]	**4** [55–58]	**2** [49, 59]	**1** [49]
Voltage (V)	**23** [23–26, 28–30, 33, 36, 38, 42, 43, 60–70]	**5** [51, 64, 66, 69, 71]	**7** [51–53, 60, 69, 71, 72]	**1** [73]	**2** [60, 69]	**1** [69]
Resistance (Ω)	**34** [22, 23, 25–29, 31–36, 38, 39, 41–48, 60, 62, 64, 65, 67, 69, 70, 74–77]	**10** [39, 44, 47, 48, 50, 51, 64, 69, 76, 77]	**5** [51–53, 60, 69]	**4** [56–58, 73]	**3** [60, 69, 76]	**2** [69, 76]
Temperature (°C)	**1** [41]			**2** [56, 58]		
Coil Material	**6** [22, 25, 27, 45, 48, 69]	**3** [48, 51, 69]	**5** [51, 52, 54, 69, 78]	**3** [56–58]	**1** [69]	**1** [69]
Device	**45** [14, 22–32, 34–47, 49, 60, 62, 64, 65, 67–70, 74–77, 79–84]	**12** [39, 44, 47, 49–51, 69, 76, 77, 79, 83, 85]	**13** [49, 51–54, 60, 69, 72, 79, 83, 86–88]	**10** [14, 55–58, 73, 84, 88–90]	**7** [49, 59, 60, 69, 76, 83, 85]	**6** [49, 69, 76, 79, 83, 91]
E-Liquid	**52** [14, 22–49, 60, 62, 64–70, 74–77, 79, 80, 83, 84, 92–97]	**19** [39, 44, 47–51, 64, 66, 69, 71, 76, 77, 79, 83, 85, 93–95]	**14** [49, 51–54, 60, 69, 71, 79, 83, 86–88, 98]	**10** [14, 55–58, 73, 84, 88–90]	**7** [49, 59, 60, 69, 76, 83, 85]	**6** [49, 69, 76, 79, 83, 91]

^a^ Abbreviations: ENDS, electronic nicotine delivery device; VOC, volatile organic compound; ROS, reactive oxygen species; PAH, polycyclic aromatic hydrocarbon; TSNA, tobacco-specific nitrosamines

**Table 2:** Number of studies reporting ENDS characteristics and measured carbonyl compounds.

Characteristic	Formaldehyde	Acetaldehyde	Acrolein	Crotonaldehyde	Acetone	Propionaldehyde	Methylglyoxal	Glyoxal	Dihydroxyacetone	Butyraldehyde (butanal)	Benzaldehyde	Isovaleraldehyde (3-methylbutanal)	valeraldehyde (pentanal)	Diacetyl
Power (W)	**23** [22–26, 28–33, 36–42, 45–49]	**22** [22, 23, 25–29, 32, 33, 35–42, 44–49]	**22** [22, 23, 25–29, 32, 33, 35–42, 44, 46–49]	**6** [36, 38, 41, 42, 47, 49]	**14** [22, 28, 29, 32, 33, 37, 40–42, 44, 45, 47–49]	**14** [22, 27, 29, 32, 33, 36, 38–41, 44, 45, 47, 49]	**6** [22, 32, 33, 41, 48, 49]	**7** [32, 33, 38, 41, 47–49]	**2** [34, 44]	**5** [29, 38, 41, 47, 49]	**4** [32, 38, 43, 47]		**5** [32, 38, 41, 45, 47]	**1** [49]
Voltage (V)	**19** [23–26, 28–30, 33, 36, 38, 42, 60–65, 69, 70]	**18** [23, 25, 26, 28, 29, 33, 36, 38, 42, 60–66, 69, 70]	**18** [23, 25, 26, 28, 29, 33, 36, 38, 42, 60–66, 69, 70]	**8** [36, 38, 42, 61, 62, 65, 69, 70]	**10** [28, 29, 33, 42, 61, 62, 65, 66, 69, 70]	**10** [29, 33, 37, 38, 61–63, 65, 69, 70]	**2** [33, 69]	**3** [33, 38, 69]		**7** [38, 61–63, 65, 69, 70]	**7** [38, 43, 61, 62, 67, 68, 70]	**4** [61–63, 70]	**6** [38, 61–63, 65, 70]	**2** [64, 69]
Resistance (Ω)	**28** [22, 23, 25, 26, 28, 29, 31–33, 36, 38, 39, 41, 42, 45–48, 60, 62, 64, 65, 69, 70, 74–77]	**30** [22, 23, 25–29, 32, 33, 35, 36, 38, 39, 41, 42, 44–48, 60, 62, 64, 65, 69, 70, 74–77]	**29** [22, 23, 25–29, 32, 33, 35, 36, 38, 39, 41, 42, 44, 46–48, 60, 62, 64, 65, 69, 70, 74–77]	**11** [36, 38, 41, 42, 47, 62, 65, 69, 70, 74, 76]	**19** [22, 28, 29, 32, 33, 41, 42, 44, 45, 47, 48, 62, 65, 69, 70, 74–77]	**19** [22, 27, 29, 32, 33, 36, 38, 39, 41, 44, 45, 47, 62, 65, 69, 70, 74, 76, 77]	**8** [22, 32, 33, 41, 48, 49, 69, 77]	**9** [32, 33, 38, 41, 47–49, 69, 77]	**3** [34, 44, 77]	**11** [29, 38, 41, 47, 49, 62, 65, 69, 70, 74, 76]	**8** [32, 38, 43, 47, 62, 67, 70, 74]	**3** [62, 70, 74]	**9** [32, 38, 41, 45, 47, 62, 65, 70, 74]	**3** [64, 69, 77]
Temperature (°C)	**1** [41]	**1** [41]	**1** [41]	**1** [41]	**1** [41]	**1** [41]	**1** [41]	**1** [41]		**1** [41]			**1** [41]	
Coil Material	**5** [22, 25, 45, 48, 69]	**6** [22, 25, 27, 45, 48, 69]	**5** [22, 25, 27, 48, 69]	**1** [69]	**4** [22, 45, 48, 69]	**4** [22, 27, 45, 69]	**3** [22, 48, 69]	**2** [48, 69]		**1** [69]			**1** [45]	**1** [69]
Device	**38** [14, 22–26, 28–32, 36–42, 45–47, 49, 60, 62, 64, 65, 69, 70, 74–77, 79, 80, 83, 84]	**38** [14, 22, 23, 25–29, 32, 35–42, 44–47, 49, 60, 62, 64, 65, 69, 70, 74–77, 79, 80, 83, 84]	**36** [14, 22, 23, 25–29, 32, 35–42, 44, 46, 47, 49, 60, 62, 64, 65, 69, 70, 74–77, 79, 80, 83]	**16** [14, 36, 38, 41, 42, 47, 49, 62, 65, 69, 70, 74, 76, 79, 83]	**24** [14, 22, 28, 29, 32, 37, 40–42, 44, 45, 47, 49, 62, 65, 69, 70, 74–77, 79, 84]	**24** [14, 22, 27, 29, 32, 36, 38–41, 44, 45, 47, 49, 62, 65, 69, 70, 74, 76, 77, 79, 84]	**6** [22, 32, 41, 49, 69, 77]	**7** [32, 38, 41, 47, 49, 69, 77]	**3** [34, 44, 77]	**11** [29, 38, 41, 47, 49, 62, 65, 69, 70, 74, 76]	**10** [32, 38, 43, 47, 62, 67, 68, 70, 74, 79]	**4** [62, 70, 74, 79]	**10** [32, 38, 41, 45, 47, 62, 65, 70, 74, 79]	**6** [38, 49, 64, 69, 77, 82]
E-Liquid	**43** [14, 22–26, 28–33, 36–42, 45–49, 60, 62, 64, 65, 69, 70, 74–77, 79, 80, 83, 84, 94, 96, 97]	**44** [14, 22, 23, 25–29, 32, 33, 35–42, 44–49, 60, 62, 64–66, 69, 70, 74–77, 79, 80, 83, 84, 94, 96, 97]	**42** [14, 22, 23, 25–29, 32, 33, 35–42, 44, 46–49, 60, 62, 64–66, 69, 70, 74–77, 79, 80, 83, 93, 94, 96]	**16** [14, 36, 38, 41, 42, 47, 49, 62, 65, 69, 70, 74, 76, 79, 83, 97]	**28** [14, 22, 28, 29, 32, 33, 37, 40–42, 44, 45, 47–49, 62, 65, 66, 69, 70, 74–77, 79, 84, 94]	**27** [14, 22, 27, 29, 32, 33, 36, 38–41, 44, 45, 47, 49, 62, 65, 69, 70, 74, 76, 77, 79, 84, 94, 97]	**8** [22, 32, 33, 41, 48, 49, 69, 77]	**9** [32, 33, 38, 41, 47–49, 69, 77]	**3** [34, 44, 77]	**11** [38, 41, 47, 49, 62, 65, 69, 70, 74, 76, 95]	**13** [32, 38, 43, 47, 62, 67, 68, 70, 74, 79, 94, 95, 97]	**5** [62, 70, 74, 79, 95]	**12** [32, 38, 41, 45, 47, 62, 65, 70, 74, 79, 95, 97]	**6** [49, 64, 69, 77, 92, 97]

### Associations between ENDS characteristics and aerosol toxicants

We further categorized articles based on their reporting of ENDS attributes and how differences in devices and operating conditions influence the presence of aerosol toxicants (Table 3); the absence of a correlation did not prevent a particular study from being included in Table 3. Below, the studies are grouped by the toxicants examined, and their main findings are summarized.

**Table 3:** Number of studies reporting associations between varying ENDS^a^ characteristics and aerosol toxicants.

Characteristic	Carbonyls	VOCs	Trace Elements	ROS and Free Radicals	PAHs	TSNAs
Power (W)	**21** [22–27, 29, 31–38, 40, 43–45, 47, 48]	**4** [44, 47, 48, 50]	**1** [52]	**3** [55–57]		
Voltage (V)	**11** [23–26, 29, 33, 38, 43, 62, 64, 70]	**2** [64, 71]				
Resistance (Ω)	**14** [22, 26, 29, 32–36, 38, 44, 45, 47, 48, 64]	**5** [44, 47, 48, 50, 64]	**1** [52]			
Temperature (°C)				**1** [56]		
Coil Material	**2** [22, 48]	**1** [48]	**1** [52]			
Device	**19** [14, 22, 23, 26, 29, 32, 34–38, 40, 44, 45, 47, 64, 79, 80, 83]	**6** [44, 47, 50, 64, 79, 83]	**6** [52, 54, 72, 79, 83, 87]	**4** [14, 55, 89, 90]	**1** [83]	**2** [79, 83]
E-Liquid	**31** [14, 23, 25, 28, 29, 33, 35, 36, 38–40, 42, 43, 46, 47, 60, 62, 64, 67, 68, 70, 74, 75, 79, 80, 83, 84, 92–94, 97]	**11** [39, 47, 50, 51, 64, 71, 79, 83, 85, 93, 94]	**8** [52, 54, 60, 79, 83, 86, 87, 98]	**9** [14, 55–58, 73, 84, 89, 90]	**4** [59, 60, 83, 85]	**3** [79, 83, 91]

^a^ Abbreviations: ENDS, electronic nicotine delivery device; VOC, volatile organic compound; ROS, reactive oxygen species; PAH, polycyclic aromatic hydrocarbon; TSNA, tobacco-specific nitrosamines

### Carbonyls/VOCs

Numerous studies have evaluated the relationship between power supplied to the atomizer heating element and aerosol carbonyl concentrations. Salamanca *et al*. [31] reported carbonyl levels of 1.20 μg/mg e-liquid consumed when an ENDS was operated at a power of 10 watts (W) and 4.43 μg/mg e-liquid with the power at 15 W. Beauval *et al*. [22] observed that carbonyl concentrations in aerosol were higher when the ENDS was operated at 30 W than at 18 W. Korzun *et al*. [27] marked propionaldehyde, acetaldehyde, acrolein, glycoaldehyde, hydroxyacetone below the limits of detection and quantification at 11, 13, and 17 W, but all carbonyls were above the limit of quantification at 24 W. El-Hellani *et al*. [40] concluded that power significantly correlated with total aldehydes in ENDS aerosol. In a particular ENDS recommended for use at 15–60 W, Uchiyama *et al*. [48] detected carbonyls at high levels at 50 W (4400 μg/15 puffs) and even higher at 60 W (6200 μg/15 puffs). Jensen *et al*. [44] identified carbonyls and VOCs in ENDS aerosol in single-puff experiments and observed increases in the measured compounds when power increased from 4 W to 6 W; the aerosol produced had a unique fingerprint that distinguished it from the unaerosolized e-liquid. Ogunwale *et al*. [29] observed that increasing ENDS power from 11.7 W to 16.6 W dramatically increased the aerosol concentrations of acetaldehyde, acrolein, formaldehyde, propionaldehyde, and butyraldehyde. Geiss *et al*. [25] observed that carbonyl production significantly increased with increasing power, with a sharp rise at 15 W and 20 W. Talih *et al*. [32] concluded that carbonyl concentrations in ENDS aerosols are correlated with both power applied per surface area of coil and liquid consumed. Vreeke *et al*. [35] observed that acrolein and acetaldehyde remained below the limit of detection when power increased from 55 W to 65 W on a sub-ohm (<1 ohm resistance) heating coil, but these toxicants increased when power increased from 9 W to 11 W on a supra-ohm (>1 ohm resistance) heating coil. Vreeke *et al*. [34] found that device temperature increases with increasing power and corresponds directly to dihydroxyacetone (DHA) production. Pankow *et al*. [50] observed that benzene concentrations in aerosol increased with increasing wattage from 6 W to 13 W on one device, but remained undetectable when wattage increased from 6 W to 20 W on a different device. Gillman *et al*. [26] observed that an increase in the efficiency of aerosol production, defined as (milligrams aerosol per puff) per watt of power applied to the coil, was associated with lower levels of aldehydes in the aerosol. Farsalinos *et al*. [24] observed that aerosol formaldehyde concentrations increased with increasing power applied to the heating coil, but also concluded that daily exposure to formaldehyde from consuming 3 grams of liquid would be 32% lower than from smoking cigarettes. Overall, the findings suggest that increased power is associated with increased carbonyl production.

The available literature suggests a similar relationship between voltage and the amount of carbonyls in ENDS aerosol. Uchiyama *et al*. [33] observed that carbonyls increased when applied voltage increased from 3.2 V to 4.4 V. Kośmider *et al*. [62] observed that PG-containing fluids were associated with significantly higher levels of formaldehyde and acetone when an ENDS was operated at 4.8 V than at 3.2 V. Sleiman *et al*. [64] observed that increasing the voltage applied to a single-coil device from 3.3 V to 4.8 V tripled total aldehyde emission rates from 53 to 165 μg/puff, and reusing the device several times increased aldehydes by more than 60%. Behar *et al*. [43] observed hydroxyacetone in ENDS aerosols at concentrations ranging from ~100 to ~10,000 μg/mL at 5 V but not at 3 V. Zhao *et al*. [71] observed that as voltage increased from 2.2 to 5.7 V, benzene increased 11-fold and toluene doubled. Similarly to increasing power, an increase in voltage has been observed to be associated with increased carbonyls in aerosol at a fixed resistance.

Suboptimal ENDS operating conditions result in what is commonly referred to as a “dry puff”, during which coil heating occurs in the absence of sufficient e-liquid to produce aerosol. Suboptimal ENDS operation can result in higher concentrations of carbonyls in aerosol. Farsalinos *et al*. [37] observed aldehydes under non–dry puff conditions but at substantially lower levels than with conventional cigarettes; however, dry puff conditions raised ENDS aerosol concentrations of formaldehyde, acetaldehyde, and acrolein by 30 to 250 times, potentially higher levels than a conventional cigarette. Available evidence seems to suggest that maintenance of optimal operation conditions (e.g., adequate wick saturation and avoiding excessive coil heating) is associated with lower levels of carbonyls.

Coil location, orientation, and resistance may also affect carbonyl production. Farsalinos *et al*. [23] observed that top-coil atomizers produce more carbonyls at higher voltages than at lower voltages, and bottom-coil atomizers produce extremely low levels of carbonyls at both high and low voltages. Gillman *et al*. [26] also observed that top-coil atomizers produce more carbonyls than bottom-coil atomizers at the same wattage. Pankow *et al*. [50] reported that benzene concentrations were largely undetectable in an ENDS with a single vertical coil with a cotton wick, but were more readily detected with a single horizontal coil with a silica wick. Vreeke *et al*. [34] observed that aerosol concentrations of DHA were directly proportional to the mass of aerosolized VG:PG, but a vertical coil produced less DHA than a horizontal coil. Stephens *et al*. [45] similarly observed more carbonyls with a top coil than a bottom coil; insufficient wick saturation was associated with coil build-up and greater carbonyl output compared to a stainless steel organic cotton coil which provided ample wick saturation with minimal coil deposits and fewer carbonyls. Son *et al*. [47] identified a top coil device generated increased concentrations of formaldehyde (4.80 μg/puff) compared to a mod device with a Clapton-style coil (0.47 μg/puff) Top-coil horizontal atomizers have the potential to generate more carbonyls compared to bottom-coil horizontal atomizers, while vertical coils produced fewer carbonyls than horizontal coils.

Several studies, but not all, have observed that carbonyl and VOC production varies by device type. Uchiyama *et al*. [33] reported significant differences in carbonyl production between different brands, whereas Flora *et al*. [83] observed low formaldehyde concentrations with low variability (0.09 to 0.33 μg/puff) across 4 of the same ENDS products. Gillman *et al*. [46] examined the formaldehyde, acetaldehyde, and acrolein output of 10 identical ENDS products noting the inherent variability observed from the devices. Goniewicz *et al*. [79] observed 4 carbonyls (among 15 analyzed) and 2 VOCs (among 11 analyzed) that were present in aerosols generated from almost all 12 ENDS brands. Bitzer *et al*. [14] observed some significant differences between several non-refillable closed-system devices and 4 carbonyls. Wagner *et al*. [85] did not detect 1,3 butadiene; isoprene; acrylonitrile; benzene; or toluene in the aerosol of 6 top-selling, commercially available ENDS. Laugesen *et al*. [80] observed across 9 ENDS brands an average formaldehyde concentration of 1.07 μg/L of vapor, 0.81 μg/L of acetaldehyde, and 1.06 μg/L of acrolein; however, the concentrations of aldehydes on average have decreased over time. El-Hellani *et al*. [40] concluded that ENDS brand significantly correlated with total aldehydes in ENDS aerosol. Vreeke *et al*. [35] reported fewer carbonyl compounds produced from a sub-ohm tank than from a supra-ohm clearomizer (atomizer and cartridge). Gillman *et al*. [26] reported that sub-ohm devices produced lower levels of carbonyls than supra-ohm devices at similar wattages. Device type has been shown to potentially impact carbonyl and VOC output.

Numerous articles have reported that e-liquid constituents impact carbonyl aerosol concentrations. Kim *et al*. [51] observed that carbonyls were present in aerosol associated with major flavoring groups but not in un-aerosolized liquid. Herrington *et al*. [94] observed that acetaldehyde and acrolein exist in ENDS aerosol but not in e-liquid solutions. Conklin *et al*. [42] demonstrated that VG generates higher levels of formaldehyde and acrolein and PG generates higher levels of acetaldehyde. Klager *et al*. [97] observed median formaldehyde concentrations of 626 μg/m^3^ in aerosol produced with 24 e-liquid flavors. Beauval *et al*. [60] observed formaldehyde and acetaldehyde close to 1 ng/mL of emission with no substantial differences between flavorings, with the exception of acrolein concentrations from aerosol produced with “blond tobacco” flavored e-liquid. Papoušek *et al*. [93] observed that acrolein was present in higher concentrations in tobacco-flavored than in fruit-flavored e-liquid. Zhao *et al*. [71] observed that menthol flavor generated 330% more benzene and 120% more toluene than in tobacco flavor. Qu *et al*. [74] observed that the carbonyl compounds emission factors increased linearly 1.0- to 92-fold when flavor content of the e-liquid increased from 5% to 50% and concluded that most carbonyls in e-liquids were from flavorings. Allen *et al*. [92] detected diacetyl in aerosols in 39 of 51 e-liquids. Kośmider *et al*. [67] detected benzaldehyde in 108 of 145 flavored e-liquids and observed the highest levels (5.1–141.2 μg/30 puffs) in cherry-flavored products. Pankow *et al*. [50] observed that benzene concentrations in ENDS aerosol increased with the addition of benzoic acid and benzaldehyde to the e-liquid solution. Vreeke *et al*. [35] observed that e-liquid containing 10% flavoring compound was associated with increased carbonyls in aerosol compared with e-liquids containing only VG:PG. Duell *et al*. [39] observed that the concentrations of aldehydes (propionaldehyde, acetaldehyde, glycolaldehyde, and acrolein) increased as sucralose concentration in the e-liquid increased. The relative concentrations of carbonyls in the aerosol have varied from study to study [36, 38]. Reilly *et al*. [84] observed no differences in formaldehyde and acetone across the aerosols of 4 different JUUL flavors. Additionally, Mallock *et al*. [75] observed that European and American versions of JUUL pods produced similar levels of carbonyls. Available evidence suggests that the chemical constituency of aerosol is dependent upon the matrix of the e-liquid as carbonyl compounds are present in the aerosol as both flavoring agents (i.e., diacetyl) and products of thermal degradation (i.e., aldehydes).

E-liquid composition also impacts the transfer of chemicals to the aerosol. Erythropel *et al*. [68] observed that benzaldehyde carryover (i.e., percentage of chemical transferred from e-liquid to aerosol) significantly increased when PG content increased from 0% PG to 100% PG. This suggests that the concentration of toxicants in aerosol can be influenced by the bulk e-liquid solution.

A common technique for carbonyl analysis is to bubble aerosol through 2,4-dinitrophenylhydrazine (DNPH). This allows carbonyls to form carbonyl-DNPH adducts that can be detected with high performance liquid chromatography. Hemiacetals are formed through the reaction of a carbonyl with a hydroxyl, such as one on PG or VG, and become unreactive to DNPH. The stability of hemiacetal adducts formed when reactive carbonyls, such as formaldehyde, interact with VG or PG is controversial, as are the implications of the results. Though not directly observed, some investigators suggest that the stability of the carbonyl-DNPH adduct drives equilibria of hemiacetals, acetals, and free carbonyls towards that of the carbonyl-DNPH adduct and the formation of hemiacetals is reversed in DNPH solution [26]; others maintain that the formation of hemiacetals leads to a severe underestimation of the absolute amount of carbonyls [31, 99]. Since an ENDS user would inhale the hemiacetal or acetal [68], both are excluded as carbonyls in the tables.

Various ENDS operating parameters increase the concentrations of hemiacetals in aerosol. Jensen *et al*. [99] reported a significant change in the presence of formaldehyde hemiacetals as voltage increased from 3.3 V (not detected) to 5.0 V (380 ± 90 μg/10 puffs). Salamanca *et al*. [31] observed approximately 4 times the amount of formaldehyde and formaldehyde hemiacetals when a device was powered at 15 W than at 10 W. However, both power settings had the same ratio of formaldehyde to formaldehyde hemiacetals. Vreeke *et al*. [35] increased power from 55 W to 65 W for a newer generation device and identified increased formaldehyde hemiacetals. Similarly, power increases from 9 W to 11 W significantly increased formaldehyde hemiacetals. Additionally, Vreeke *et al*. [35] reported that the addition of triacetin, a common flavor additive, increased levels of formaldehyde hemiacetals. Duell *et al*. [39] observed that formaldehyde hemiacetals increased with increasing concentrations of sucralose added to the e-liquid. Erythropel *et al*. [68] reported carryover rates for both aldehydes and corresponding acetals to be 50–80% of the original concentration in e-liquid. Power and voltage have been demonstrated to increase formaldehyde hemiacetals in aerosol which can be further influenced by e-liquid composition.

### Trace elements

Important relationships have been observed between trace elements in ENDS aerosols and variations in power, device, air-flow, coil material, and e-liquid. Zhao *et al*. [52] observed that when power was increased on an open-system device from 20 W to 40 W, median arsenic (As), chromium (Cr), Cu, iron (Fe), manganese (Mn), nickel (Ni), Pb, antimony (Sb), tin (Sn), and Zn concentrations increased 14, 54, 17, 30, 41, 96, 14, 81, 631, and 7-fold, respectively. Zhao *et al*. [52] observed that open-system ENDS with kanthal (Fe, Cr, and aluminum [Al] alloy) and stainless steel coils had consistently higher Fe and Ni levels. Goniewicz *et al*. [79] identified Cd, Ni, and Pb in all vapors generated from 12 different first-generation ENDS models. Prokopowicz *et al*. [72] detected Pb in the aerosol of 2-tank-system ENDS. Ting *et al*. [54] identified 5% of ENDS and e-liquid combinations tested emitted Cr at levels that exceeded permissible daily exposure limits. Williams *et al*. [87] analyzed disposable ENDS and observed that aerosols generated at low and high air-flow rates produced the same pattern of elements, although the total element concentration decreased at the higher air-flow rate. In this study, at least 35 trace elements were identified in ENDS aerosol; Si was the dominant element, but other elements such as calcium (Ca), sodium (Na), Cu, magnesium (Mg), Sn, Pb, Zn, boron (B), selenium (Se), Al, Fe, germanium (Ge), Sb, Ni, and strontium (Sr) were present in most ENDS aerosols, with relative concentrations varying by ENDS model. Beauval *et al*. [60] evaluated 6 e-liquid refills and observed that only Cd, Cr, and Sb were present in aerosol, with concentrations up to 0.14, 3.4, and 0.47 pg/mL puff, respectively. The concentrations of metals varied minimally with the presence or absence of flavoring and nicotine. In contrast, Mikheev *et al*. [86] observed that that As, Cr, Ni, Cu, Sb, Sn, and Zn levels in ENDS aerosol varied widely across nicotine- and non-nicotine-containing flavors. Liu *et al*. [98] observed certain As species significantly increased in concentration from e-liquids to aerosol. Not all published articles have detected trace elements. Flora *et al*. [83] did not detect As and cadmium (Cd) in ENDS aerosol across 4 commercial e-cigarette non-refillable closed-system products. The ten most examined elements in descending order were Cu, Ni, Zn, Pb, Cd, As, Fe, Cr, Sn, and Al.

### ROS and free radicals

The concentration of free radicals in aerosol has been reported to vary by power and associated coil temperature. Bitzer *et al*. [56] observed increases in free radicals with increases in coil temperature from 100 to 300 °C and power from 10 W to 50 W. Haddad *et al*. [55] observed that ROS flux increased 3-fold when the power increased from 5 W to 11 W on a single coil device, and increased approximately 7-fold when the power increased from 100 W to 200 W on an octuple coil device. Son *et al*. [57] observed that a device formed more hydroxyl radicals at 31.3 W than at 6.4 W. Free radicals have been demonstrated to increase with an increase in power.

The effects of device type on free radicals have been examined. Bitzer *et al*. [14] observed that free radicals are produced by non-refillable closed-system ENDS. Shein *et al*. [89] observed similar amounts of free radicals in aerosols using standard wicking and heating, a mesh coil, and an air control. Hasan *et al*. [90] found free radical concentration from two closed-system ENDS were significantly lower than conventional tobacco cigarettes, but the oxidative potency from the free radicals originating from the ENDS was much higher. No clear relationship between free radicals and device design has been observed.

The composition of e-liquid affects the concentrations of free radicals in aerosol. Bitzer *et al*. [56] observed increases in free radicals with changing VG:PG ratios from 100:0 to 0:100. Son *et al*. [57] observed that VG- and VG:PG-based e-liquids formed twice as many radicals than exclusively PG-based e-liquids; specifically, 90- and 170-mL puffs (3.8 s each) generated 4.1- and 10.3-fold more hydroxyl radicals than a 35-mL puff (also 3.8 s). Reilly *et al*. [84] analyzed aerosol from JUUL and observed that free radicals were 2.95 ± 0.81 nmol/g for tobacco flavor at a 70:30 VG:PG ratio; the concentration was higher (3.01 ± 0.28 nmol/g) when the e-liquid composition was 70:30 VG:PG without flavoring. Additionally significance was reached when the e-liquid was 40:60 VG:PG without flavoring (4.68 ± 0.58 nmol/g), and increased further to 4.78 ± 0.73 nmol/g when citral was added to the 40:60 VG:PG base. Goel *et al*. [73] generated aerosols from 3 different e-liquids generated by a tank system and estimated free-radical production to be 10.3, 4.0 and 2.5 × 10^13^ radicals per puff of menthol, citrus, and tobacco flavors, respectively. Bitzer *et al*. [58] analyzed the free radicals generated from 49 commercially available e-liquids and observed that nearly 43% of the flavors resulted in significant increases in radical production compared with the base VG:PG (40:60) mixture, but the amount varied greatly among the flavors; the flavorings dipentene, ethyl maltol, citral, linalool, and piperonal promoted radical formation in a concentration-dependent manner, whereas ethyl vanillin inhibited radical formation. Overall, ROS and free radicals in ENDS aerosol vary by e-liquid composition, but the precise relationship has yet to be determined.

### PAHs

PAH concentrations in aerosol vary with device and e-liquid but are generally low if detected. Flora *et al*. [83] analyzed aerosol from 4 commercially available e-cigarette products and observed that benzo[a]pyrene was below the limit of detection of 10 ng/device. Similarly, Wagner *et al*. [85] did not detect benzo[a]pyrene in the aerosol of 6 top-selling commercially available ENDS. Beauval *et al*. [60] systematically detected naphthalene and acenaphthylene in ENDS aerosol at low concentrations (up to 4.10 and 0.37 pg/mL puff, respectively). Eddingsaas *et al*. [59] analyzed aerosols created from vanilla, cinnamon, and mango e-liquids and observed cadalene in the mango flavor. Available evidence suggests that PAH concentrations are low which is consistent with the knowledge that PAHs are primarily products of combustion [100] and ENDS operate at lower temperatures than combusted tobacco products.

### TSNAs

Studies of different ENDS products have observed low or undetectable levels of TSNAs. Flora *et al*. [83] did not detect TSNAs (NNN and NNK) in aerosols from 4 commercially available ENDS products. Goniewicz *et al*. [79] analyzed 12 ENDS products and observed NNN concentrations ranging from not detected to 4.3 ± 2.4 ng and NNK from not detected to 28.3 ± 13.2 per 150 puffs. Farsalinos *et al*. [91] observed that no TSNAs were above the level of detection for aerosols generated from 3 tobacco-flavored e-liquids aerosolized by a second-generation ENDS. Though TSNAs are carcinogenic and their concentrations eclipse many other carcinogenic compounds in conventional cigarettes [101], they are less prevalent in ENDS.

### Additional articles

Garcia-Gomez *et al*. [102] analyzed the aerosol of an ENDS through secondary electrospray ionization mass spectrometry, which identified more than 250 chemical species, including alkaloids and various flavoring compounds. The authors noted that some substances increased with increasing power of the device. Behar *et al*. [103] found cinnamaldehyde in 20 (51%) of 39 refill fluids, and aerosols contained several different constituents at 5 V than at 3 V. Soussy *et al*. [104] reported that emissions of 5-hydroxymethylfurfural and furfural were correlated with device power and sweetener concentration in the e-liquid. These observations support the notion that power and voltage influence aerosol toxicant concentration.

Lee *et al*. [105] detected more carbonyls in ENDS aerosol generated from tobacco flavors than from menthol. Kośmider *et al*. [28, 70] observed that aerosol carbonyls varied by puffing protocol when different e-liquid nicotine concentrations were assessed. Uchiyama *et al*. [106] observed large variations in carbonyl concentrations between and within unnamed devices. El-Hage *et al*. [107] reported the transfer of pyrazines, a class of flavoring compounds, from e-liquid to aerosol. Rawlinson *et al*. [108] identified the chemical profiles of aerosols generated from 4 e-liquids contained between 30–90 compounds mostly characterized as “unknown” exceeding the 5 ng/puff threshold which may include PAHs.

El-Hellani *et al*. [109] focused on how device modifications such as power, coil material, and coil geometry may affect the emission of small gasses, notably carbon monoxide; they reported that power and coil material significantly affect analyzed chemical species, with nickel wire being the most reactive. Wang *et al*. [110] reported a device-independent study wherein various ratios of VG and PG were aerosolized at a range of temperatures, yielding carbonyl compounds. VG generated much higher concentrations of formaldehyde than PG; acrolein was detected only when VG was present in the solution and the temperature was ≥270 °C. Saliba *et al*. [111] explored the device-independent effect of the metal coil on subsequent carbonyl production. The wires had a catalytic impact on carbonyls, producing species at 250 °C, as opposed to 460 °C without wire. Moreover, the investigators found the material and age of the wire had a strong correlation with carbonyl production, with new nichrome (Ni and Cr alloy) being the least reactive and old nichrome being the most reactive. Williams *et al*. [112, 113] reported a comprehensive metal analysis of ENDS aerosol the sources of the metals to be the metal components of the heating elements such as coils, wires, or solder joints.

Belushkin *et al*. [114] completed a comprehensive toxicant panel of 34 ENDS and 57 e-liquids. Benzene was identified in some high power mod systems, but was not as prevalent in non-refillable closed systems. Carbonyl emissions were lower in closed systems than open systems. Particularly noted, formaldehyde increased with the depletion of e-liquid in the reservoir, starting at approximately 50% capacity. Trace elements, TSNAs, and PAHs were seldom above the limit of quantification.

### Risk of bias assessment

Table 4 demonstrates that the analytical techniques used to assess major chemical groups of aerosol toxicants have been largely replicated.

**Table 4:** Risk of bias determined by analytical technique utilized by studies and number of studies of measured major chemical groups in aerosol.

Technique	Carbonyls	VOCs^a^	Trace Elements	ROS and Free Radicals	PAHs	TSNAs
Group A						
HPLC	**41** [14, 22–26, 28, 30–33, 36–38, 40, 41, 45–48, 60–65, 67, 69, 70, 74–76, 79, 80, 82–84, 97, 106, 110, 111, 114]	**3** [47, 50, 69]				
GC-FID	**3** [39, 68, 92]	**2** [39, 71]				
TD-GC-MS	**2** [94, 95]	**4** [50, 64, 94, 95]				
GC-MS	**11** [29, 33, 34, 39, 41–43, 69, 81, 93, 105]	**13** [39, 48, 49, 51, 59, 69, 76, 79, 83, 85, 93, 105, 114]			**7** [49, 59, 69, 76, 83, 85, 114]	
EPR				**8** [14, 56, 58, 73, 84, 88–90]		
ICP-MS			**11** [49, 52–54, 60, 69, 78, 79, 83, 86, 114]			
ICP-OES			**4** [51, 87, 112, 113]			
SF-ICP-MS			**1** [71]			
UPLC-MS/MS						**1** [91]
UPLC-MS						**1** [79]
LC-MS/MS						**5** [49, 69, 76, 83, 114]
SPME GC-MS	**1** [96]					
Group B						
NMR	**5** [27, 34, 35, 39, 44]	**2** [39, 44]				
GC-MS/MS					**1** [60]	
Group C						
PTR-MS	**1** [66]	**1** [66]				
GC-FID/TOFMS	**1** [108]	**1** [108]			**1** [108]	
HPLC-HR-MS	**1** [77]	**1** [77]				
SESI-HR-MS	**1** [102]	**1** [102]				
Fluorescence^b^				**1** [55]		
Fluorescence^c^				**1**[57]		
electrothermal AA			**1** [72]			
AA			**1** [88]			
HPLC-ICP-MS			**1** [98]			
EDXRF			**1** [105]			
FT-ICR-MS	**1** [42]					
Total Individual Studies						
	**60**	**24**	**20**	**10**	**9**	**7**

^a^Abbreviations: VOCs, volatile organic compound; ROS, reactive oxygen species; PAH, polycyclic aromatic hydrocarbon; TSNA, tobacco-specific nitrosamines; HPLC, high performance liquid chromatography; GC-FID, gas chromatography–coupled flame ionization detector; TD-GC-MS, thermal desorption gas chromatography mass spectrometry (MS); GC-MS, gas chromatography MS; EPR, electronic paramagnetic resonance spectroscopy; ICP-MS, inductively coupled plasma MS; ICP-OES, inductively coupled plasma optical emission spectrometry; SF-ICP-MS, sector field inductively coupled plasma MS; UPLC-MS/MS, ultra performance liquid chromatography tandem MS; UPLC-MS, ultra performance liquid chromatography MS; LC-MS/MS, liquid chromatography tandem MS; SPME GC-MS, solid phase microextraction gas chromatography MS; NMR, nuclear magnetic resonance spectroscopy; GC-MS/MS, gas chromatography tandem MS; PTR-MS, proton transfer reaction MS; GC-FID/TOFMS, gas chromatography–coupled flame ionization detector coupled time of flight MS; HPLC-HR-MS, HPLC high resolution-MS; SESI-HR-MS, secondary electrospray ionization HR-MS; electrothermal AA, electrothermal atomic absorption spectroscopy; AA, atomic absorbance spectrometry; EDXRF, energy dispersive X-ray fluorescence spectroscopy; FT-ICR-MS, Fourier transform ion cyclotron resonance MS

^b^ flurorescence SpectraMax M5 microplate

^c^ fluorescence Synergy 4 multidetection microplate

## Discussion

We identified 92 articles reporting ENDS characteristics and aerosol toxicants, the majority of which used analytic techniques replicated by other researchers and many of which attempted to define relationships between changes in ENDS characteristics and aerosol constituents. Device characteristics relevant to the current review of ENDS are power, voltage, resistance, temperature, coil material, device, and e-liquid. The major chemical constituents were differentiated into carbonyls, VOCs, trace elements, ROS and free radicals, PAHs, and TSNAs.

The ENDS industry evolves rapidly, and contemporary scientific observations may not maintain relevancy over time. Williams *et al*. [115] characterized ENDS manufactured from 2011 to 2017, noting significant variation in atomizer design over time within and between brands and indicating the importance of acknowledging and monitoring device design changes. Despite these rapid changes, the extant literature provides insight into ways that the technology could be improved and aerosol toxicants minimized either by the actions of ENDS manufacturers or through regulation, thereby reducing potential adverse health impacts.

First, improving the safety profile of e-liquids would be a critical first step in manufacturing and regulation to reduce the likelihood of adverse health consequences from ENDS use. The risk associated with inhaling ENDS aerosol is associated with “unintentional” contaminants (i.e., impurities), “intentional” constituents (i.e., PG, VG, and flavorings), and the conversion of unintentional and intentional constituents into new chemical species during aerosolization. The literature suggests that numerous new chemicals are created during aerosolization that are not present in the native e-liquid [51, 59, 94]. Part of the complexity of aerosol and e-liquid composition analysis arises from the addition of flavoring compounds. Flavorings are an integral aspect of the customization of ENDS. Almost all ENDS users report using a flavored e-liquid; only 1% of users prefer the chemically simpler unflavored e-liquid [116]. E-liquid manufacturers are not mandated to list all flavoring additives as a part of the ingredients list. Moreover, many flavorings have not been tested for toxicity or potential harm caused by chemical transformations through heated aerosolization and inhalation. A handful of chemical agents used in the flavoring of e-liquid have been specifically investigated. Diacetyl and acetyl propionyl, which produce buttery or creamy flavors, have received attention for associations with bronchiolitis obliterans (i.e., “popcorn lung”) among microwave popcorn industry workers [117, 118] and have been investigated in e-liquid flavoring [82, 92]. Cinnamaldehyde has been observed to be cytotoxic [103]. Additionally, ethyl maltol and maltol have both been observed to interact with Cu or Fe to generate hydroxypyranone complexes that can promote the generation of radicals [58]. Although not within the scope of this review, cell toxicity and animal studies have been and can be leveraged to determine toxicity associated with e-liquids. Identification and characterization of toxicants both in the native e-liquid and those produced through aerosolization should be required of manufacturers. Regulatory agencies should provide guidance on safe levels of exposure in order to facilitate industry adherence.

Second, the relationship between power, resistance, and voltage (*power = voltage*^*2*^*/resistance*) affects the potential risk associated with the use of ENDS. Aerosol toxicants generally increase with increased power and voltage applied to the metal coil for heating. Power per surface area of coil may be a stronger predictor of toxicant production in aerosol than power alone [32]. Sub-ohm coils were introduced into the market to improve aerosol production through rapid coil heating; however, this phenomenon occurs at the expense of battery life. Sub-ohm devices have enabled ENDS to be operated at high wattages. Manufacturer-recommended operating ranges reach into the triple digits but produce less carbonyl than supra-ohm coils at the same power setting [26]. Researchers have suggested for manufacturers to limit power flow to coils to reduce exposure to formaldehyde, acetaldehyde, and acrolein [114]. More research needs to be conducted to understand the impact of newer-generation coils (e.g., mesh, Clapton, alien, staple) on toxicant production. Researchers can advance the science through consistent reporting of power, resistance, and voltage as well as use of standardized units of measure.

Third, atomizer design, specifically as it relates to the adequate supply of e-liquid to a heating coil, is critical to avoid the observed phenomenon of higher toxicant production from coil overheating caused by a deficiency of e-liquid for aerosolization (i.e., dry puff). This appears to be more common among top-coil designs due to inadequate wicking and could be mitigated with improved wicking to the top coil or the use of bottom, vertically oriented coils. In addition to coil location, wicking material has also evolved. In earlier generations of ENDS, the wick was primarily silica-based. With newer generations, the ENDS industry has begun to explore other wicking materials such as cotton, stainless steel, bamboo, and ceramic. Various wicking materials will deliver e-liquid to the coil at different rates; to avoid the dry puff phenomenon, these rates should be taken into consideration to ensure that the coil is properly saturated with e-liquid at all operating temperatures. The viscosity and density of the e-liquid will also determine its mobility, capillary action, and delivery to the coil, influencing the likelihood of a dry puff. Some ENDS manufacturers have included microprocessors that will deactivate heating once a certain temperature threshold is reached (e.g. >350 °C), which may minimize toxicant production from a dry puff without compromising user experience by suppressing aerosol production.

Fourth, atomizer heating elements are an identified source of aerosol metals, and metals used for construction of coils and other components (i.e., clamps, wicks, and soldering) should be selected to minimize metal breakdown and leaching. Commonly identified metals were Cu, Pb, Ni, Sn, and Zn. Coils are traditionally Ni, Ti, kanthal (Fe, Cr, and Al), stainless steel, and nichrome (Ni and Cr), whereas solder joints are Sn or Pb and wires are Cu [87]. The resistance of the alloys kanthal and nichrome remain relatively constant with changing temperature, making them ideal for use in wattage-control mode (i.e., fixed power supply with variable coil temperature); stainless steel and titanium are better suited for temperature-controlled devices (fixed coil temperature and variable power supply). High-quality metals with few defects might be less likely to leach under repeated heating-cooling cycles; coupled with timely replacement of coils, this could lead to reduced metal concentrations in aerosol. The literature suggests that the age of the coil influences carbonyl production [111], with higher carbonyl emissions associated with older devices or components [64]. To minimize potential toxicant exposure, ENDS manufacturers should select high-quality metals, minimize soldering parts, and recommended timing of coil replacement as a function of coil use.

Our review has several limitations. First, due to the variability in reporting we were unable to quantitatively estimate and provide confidence intervals around the relationships between device operative parameters (e.g., temperature, wattage) and resulting toxicant levels in the aerosol. Second, we did not specifically assess the influence of human puffing topography variability on toxicity. The ability to quantitate “estimates of effect” would become increasingly possible if the market were to contract, products were regulated, devices were standardized for clinical therapeutic interventions, and researchers were to rigorously report operating parameters to allow comparison across studies.

## Conclusion

Toxicants with potential risk to health exist in aerosol produced by ENDS. Available literature suggests that ENDS can be designed to minimize exposure to potentially harmful aerosol toxicants. Minimizing toxicants in aerosol through consideration and optimization of design could serve to reduce health risks associated with the use of ENDS as part of a comprehensive approach to reducing tobacco harm on the population level.

## Supporting information

S1 Checklist PRISMA checklist. (DOC)

S1 Table Summary of studies reporting ENDS and analyzing ENDS aerosols. (DOCX)
