# Supplementary material for: Electronic nicotine delivery system design and aerosol toxicants: A systematic review
Source: PLoS One. 2020 Jun 4;15(6):e0234189. doi: 10.1371/journal.pone.0234189 (PMC7272070; doi:10.1371/journal.pone.0234189)
Supplement: S1 Table — (DOCX) [file pone.0234189.s002.docx]

**mS2 Table**. Summary of Studies Reporting ENDS^a^ and Analyzing ENDS Aerosols

| Carbonyls/VOCs | | |
| --- | --- | --- |
| Author | **Year** | **Major Conclusions/Observations** |
| Bitzer *et al.* [14] | 2019 | Carbonyl concentration in aerosol was affected by puff duration and volume |
| Beauval *et al.* [22] | 2019 | Carbonyl in aerosols was affected by puffing regimen and ENDS device |
| Farsalinos *et al.* [23] | 2018 | New-generation ENDS emitted minimal carbonyls at high and low power under realistic conditions |
| Geiss *et al.* [25] | 2016 | Carbonyl production significantly increased at power over 15 W; authors concluded that power above 20 W is unrealistic due to negative experiences for the user |
| Farsalinos *et al.* [24, 37] | 2015, 2017 | ENDS produced high levels of carbonyls only in suboptimal operating conditions, and carbonyl emissions were minimal even with high-power ENDS under normal operating conditions |
| Gillman *et al.* [26] | 2016 | Power increases were associated with both increased and decreased carbonyl production, depending upon device |
| Korzun *et al.* [27] | 2018 | Airflow rates significantly impacted carbonyl concentrations in aerosol, and increased airflow increases e-liquid consumption |
| Kośmider *et al.* [28] | 2018 | Aerosol carbonyls varied by puffing topography when e-liquid nicotine concentrations change |
| Ogunwale *et al*. [29] | 2017 | Newer-generation ENDS produced more carbonyls specifically at a battery power output of ≥11.7 W |
| Salamanca *et al.* [30] | 2018 | Carbonyl concentrations in aerosol exceeded thresholds for workplace safety regulations; in a reexamination of their previous study[99] to address critiques, continued to detect formaldehyde and related hemiacetals above OSHA guidelines |
| Salamanca *et al.* [31] | 2017 | F/ormaldehyde hemiacetals were present at hi 2concentrations than free formaldehyde in aerosol; formaldehyde-derived hemiacetals formed in relation to power settings |
| Talih *et al.* [32] | 2017 | Carbonyls in aerosol correlated with power when standardized by surface area of the ENDS |
| Uchiyama *et al*. [33] | 2016 | Carbonyl concentrations in aerosols varied widely between and within devices |
| Vreeke *et al.* [34] | 2018 | Higher concentrations of carbonyls were observed in aerosol produced by coils in horizontal orientation than vertical orientation |
| Vreeke *et al.* [35] | 2018 | Triacetin, a popular e-liquid flavoring agent, increased the production of carbonyls in aerosol; formaldehyde hemiacetals change with varying power settings, e-liquids, and devices |
| Farsalinos *et al.* [36] | 2018 | Using e-liquids previously evaluated by these investigators[38] aerosols produced by two different ENDS had significantly lower carbonyl concentrations, with flavorless e-liquids having the lowest concentrations |
| Khlystov *et al.* [38] | 2016 | Carbonyls in aerosol were exponentially dependent on the concentration of flavoring compounds |
| Duell *et al.* [39] | 2019 | The presence of sucralose in e-liquids increased the presence of aldehydes with aerosolization; changes in e-liquid composition resulted in differences in formaldehyde hemiacetal concentration |
| El-Hellani *et al.* [40] | 2018 | Carbonyl concentrations depended on device power settings |
| Talih *et al.* [41] | 2019 | Carbonyl in aerosol produced by a JUUL device was similar to those in “closed” (non-refillable) systems |
| Conklin *et al.* [42] | 2018 | VG:PG ratios and flavorants influenced carbonyl aerosol concentrations |
| Behar *et al.* [43] | 2018 | Carbonyls were produced from flavoring compounds in 39 e-liquids at higher ENDS operating voltages |
| Jensen *et al.* [44] | 2017 | Carbonyls/VOCs were detectable in single-puff experiments using nuclear magnetic resonance; the same hemiacetals of a simple e-liquid mixture of VG and PG are present at different power settings |
| Stephens *et al.* [45] | 2019 | Increased power increased carbonyl output |
| Gillman *et al.* [46] | 2020 | Device variability overshadowed flavor-dependent carbonyl output |
| Son *et al*. [47] | 2020 | Carbon monoxide and carbonyl emissions were more heavily dependent on flavoring components rather than PG or VG |
| Uchiyama *et al.* [48] | 2020 | Carbonyls generated from thermal decomposition were detected in concentrations higher than that of conventional cigarettes |
| Nicol *et al.* [49] | 2020 | Fewer carbonyls and VOCs were reported with a new device utilizing a stainless steel mesh capillary-induced wicking and heating technology rather than ENDS with a standard wick and coil [69] |
| Pankow *et al.* [50] | 2017 | Benzene was produced by open-tank ENDS aerosolizing e-liquid solutions containing PG, VG, benzaldehyde, nicotine, and benzoic acid, but not by JUUL |
| Kim *et al.* [51] | 2018 | Aerosol produced from sweet e-liquids increased the cariogenic (i.e., cavity-causing) potential of vaping |
| Beauval *et al*. [60] | 2017 | Aerosol carbonyls were present in lower concentrations than found in cigarettes |
| Jo *et al.* [61] | 2016 | Carbonyl concentrations were higher in aerosol relative to their initial concentration in e-liquids |
| Kośmider *et al.* [62] | 2014 | Increasing ENDS operating voltages correlated with increased production of formaldehyde, and PG-based solutions produce the most carbonyls |
| Lee *et al*. [63] | 2018 | Carbonyls were present in both e-liquid and aerosols |
| Sleiman *et al.* [64] | 2016 | Aerosol concentration of carbonyls increased with voltage applied to a single-coil device more than in double-coil devices, with higher voltage settings on a single-coil device, or with device reuse |
| Talih *et al.* [65] | 2016 | “Direct dripping” produced carbonyls at levels that exceeded those reported for conventional ENDS; increasing the inter-drip interval resulted in lower nicotine yield and greater carbonyl production |
| Blair *et al.* [66] | 2015 | Carbonyls/VOCs in aerosol varied with brand, battery voltage, and e-liquid flavoring |
| Kośmider *et al.* [67] | 2016 | The concentration of benzaldehyde was highest in cherry-flavored e-liquids |
| Erythropel *et al.* [68] | 2018 | Select flavorings and PG reacted to form flavor aldehyde PG acetals, 50-80% of which were carried over into the ENDS aerosol |
| Margham *et al.* [69] | 2016 | Aerosol was less complex and contained fewer toxicants than conventional cigarette smoke |
| Kośmider *et al.* [70] | 2020 | Exposure of formaldehyde and acetaldehyde were higher in low-nicotine than high-nicotine e-liquids with fixed power |
| Zhao *et al.* [71] | 2018 | Aerosol concentrations of carbonyls and VOCs depended on e-liquid, puffing topography, and ENDS type and voltage settings |
| Qu *et al.* [74] | 2018 | Fruit e-liquid flavorings produced the highest quantities of formaldehyde in aerosol |
| Mallock *et al.* [75] | 2020 | The European version of JUUL had increased vaporization compared to the American version, though it was not associated with increased emission of carbonyls |
| Rudd *et al.* [76] | 2020 | Carbonyls and VOCs were below the limits of detection and quantification |
| Li *et al.* [77] | 2020 | The most prevalent compounds in ENDS aerosol were formaldehyde, hydroxyacetone, acetaldehyde, lactaldehyde, acrolein, and dihydroxyacetone |
| Goniewicz *et al*. [79] | 2014 | Aerosol toxicants were present in concentrations 9 to 450 times lower than those observed in conventional tobacco cigarettes |
| Laugesen *et al.* [80] | 2015 | Aerosols of later generations contained 200 times fewer toxicants than a conventional cigarette and 73% less aldehyde than early-generation ENDS |
| Pellegrino *et al.* [81] | 2012 | Toxicants were present in similar proportions in e-liquid and aerosol, with fewer particulate emissions than conventional tobacco cigarettes |
| Farsalinos *et al.* [82] | 2015 | Almost one-half of sweet-flavored e-liquids containing acetyl propionyl and diacetyl exposed users to levels higher than those generally accepted as safe |
| Flora *et al.* [83] | 2016 | Aerosol toxicants were below occupational exposure limits |
| Reilly *et al.* [84] | 2018 | Aerosol carbonyls produced by JUUL increased with increasing amounts of PG and citral, a flavoring agent |
| Wagner *et al.* [85] | 2018 | VOCs were not detected in aerosols of 6 top-selling, commercially available ENDS |
| Allen *et al.* [92] | 2016 | Most aerosols produced with 51 fruit, cocktail, or candy flavored e-liquid contain carbonyls |
| Papoušek *et al.* [93] | 2014 | Acrolein was observed in aerosol, but acrylamide is only present in tobacco smoke |
| Herrington *et al.* [94] | 2015 | New carbonyls and VOCs formed through the process of e-liquid aerosolization |
| Kim *et al.* [95] | 2015 | VOCs were produced through e-liquid aerosolization |
| Sala *et al.* [96] | 2017 | New carbonyl compounds were formed during the vaping process and depend on device heating and puffing topography |
| Klager *et al.* [97] | 2017 | When ENDS user settings were imitated, aerosols had at least one carbonyl compound known to be an HPHC |
| Jensen *et al.* [99] | 2015 | Formaldehyde hemiacetals remain undetected through traditional analytical methods, but were unstable and act as “formaldehyde-releasing agents” |

| Trace Elements | | |
| --- | --- | --- |
| Author | **Year** | **Description** |
| Nicol *et al.* [49] | 2020 | The device tested had less Zn than conventional tobacco cigarettes and a compared study [69] though fluctuating levels in air blanks masked true levels |
| Kim *et al.* [51] | 2018 | Ca, Mg, Si, Cu, and Fe were present in the aerosol of a sub-ohm ENDS |
| Zhao *et al.* [52] | 2019 | ENDS power settings and device type affect metal release into aerosol; greater power was associated with greater release in closed-system ENDS, and open systems have higher metal levels in their aerosol than closed systems |
| Palazzolo *et al.* [53] | 2016 | Al, As, Cd, Cu, Fe, Mn, Ni, Pb, and Zn were detected at lower levels in aerosol than in conventional tobacco smoke |
| Ting *et al.* [54] | 2020 | Cr, Ni, Pb, and Cd were present in ENDS aerosol; 5% of samples contained Cr at levels that exceeded permissible daily exposure |
| Beauval *et al*. [60] | 2017 | Lower concentrations of metals were observed in ENDS aerosol than in conventional cigarette smoke |
| Margham *et al.* [69] | 2016 | Cr, As, Ni, Zn, Fe, and Cu were present in aerosol |
| Zhao *et al.* [71] | 2018 | A variety of trace elements, predominantly B, Na, AI, Cu, and Zn, were present in ENDS aerosol |
| Prokopowicz *et al.* [72] | 2018 | Cd was not detected in ENDS aerosols and Pb was only detected in 2 aerosols produced by 2-tank ENDS, whereas these metals were consistently detected in conventional cigarette smoke |
| Olmedo *et al.* [78] | 2018 | Cr, Ni, Pb, Mn, and Zn were present in aerosol from 56 devices |
| Goniewicz *et al*. [79] | 2014 | Cd, Ni, and Pb were present in ENDS aerosol |
| Flora *et al.* [83] | 2016 | As and Cd were not detected in aerosol |
| Mikheev *et al.* [86] | 2016 | Sb, Sn, As, Cr, Ni, Cu, and Zn displayed large levels of variation amongst nicotine and non-nicotine containing flavors. |
| Williams *et al.* [87] | 2017 | Si was the dominant element in aerosol; other trace elements that appeared frequently were K, Ca, Cu, Sn, B and Zn |
| Lerner *et al.* [88] | 2015 | Cu in ENDS aerosol was 6.1 times higher per puff than in conventional cigarette smoke |
| Liu *et al.* [98] | 2020 | Inorganic As was present in ENDS aerosol |

| ROS & Free Radicals | | |
| --- | --- | --- |
| Author | **Year** | **Description** |
| Bitzer *et al.* [14] | 2019 | Free radical production was affected by puff duration and puff volume |
| Haddad *et al.* [55] | 2019 | ROS flux increased with power per surface area of coil and VG-based e-liquids in aerosols, produced by supra-(>1 Ω resistance) and sub-(<1 Ω resistance)ohm electronic cigarettes with different power settings, liquid compositions, and coil design |
| Bitzer *et al.* [56] | 2018 | Free radicals formation increased as temperature, wattage, and the ratio of PG to VG increased |
| Son *et al.* [57] | 2019 | Increasing puff volume, power, VG levels, and flavoring elements increased the presence of hydroxyl radicals |
| Bitzer *et al.* [58] | 2018 | E-liquid flavorings correlated with free radical emissions by enhancing or inhibiting free radical formation |
| Goel *et al.* [73] | 2015 | Short-lived, highly reactive free radicals were in aerosol |
| Reilly *et al.* [84] | 2018 | No differences in free radicals between 4 different JUUL flavors; when JUUL e-liquids was replaced with nicotine-free 40:60 VG:PG, oxidant production increased up to 190%, and the addition of citral increased levels further |
| Lerner *et al.* [88] | 2015 | Device construction influenced the quantity and type of radical produced |
| Shein *et al.* [89] | 2019 | Electronic cigarettes and heat-not-burn products released fewer free radicals in aerosol than conventional cigarettes |
| Hasan *et al.* [90] | 2020 | Free radicals were present in ENDS aerosol in lower concentrations than conventional cigarettes, though their oxidative potency was higher |

| PAHs | | |
| --- | --- | --- |
| Author | **Year** | **Description** |
| Nicol *et al.* [49] | 2020 | PAHs were detected in low levels in both the ENDs samples and blanks |
| Eddingsaas *et al.* [59] | 2018 | Cadalene was present in the aerosol of mango flavored e-liquid |
| Beauval *et al.* [60] | 2017 | Acenaphthylene and naphthalene were present in aerosol concentrations above the limit of quantification, whereas the majority of PAHs were not detected above this limit; although 10 of 16 PAHs targeted were detected in the e-liquid |
| Margham *et al.* [69] | 2016 | PAH species were either below the limit of detection or not detectable, with the exception of chrysene |
| Rudd *et al.* [76] | 2020 | Benzo[a]pyrene was below the limit of detection |
| Flora *et al.* [83] | 2016 | Benzo[a]pyrene was not detected in 4 commercial ENDS with tobacco or menthol flavor |
| Wagner *et al.* [85] | 2018 | Benzo[a]pyrene was not detected in commercially available devices and e-liquids |

| TSNAs | | |
| --- | --- | --- |
| Author | **Year** | **Description** |
| Nicol *et al.* [49] | 2020 | NNN was below the limit of quantification |
| Margham *et al.* [69] | 2016 | The TSNA concentration in ENDS aerosol was 99.8% lower than in a reference cigarette |
| Goniewicz *et al*. [79] | 2014 | TSNAs were identified in ENDS aerosol |
| Flora *et al.* [83] | 2016 | TSNAs were not present in amounts above the limit of detection or quantification |
| Rudd *et al.* [76] | 2020 | TSNAs were below the limits of detection |
| Farsalinos *et al.* [91] | 2015 | TSNA levels in the aerosol can be accurately determined from concentrations found in the e-liquid |

^a^Abbreviations: ENDS, electronic nicotine delivery device; VOCs, volatile organic compounds; HPHC, harmful and potentially harmful constituents; PG, propylene glycol; VG, vegetable glycerin (glycerol); OSHA, Occupational Safety and Health Administration; Al, aluminum; As, arsenic; Cd, cadmium; Cu, copper; Fe, iron; Mn, manganese; Ni, nickel; Pb, lead; Zn, zinc; B, boron; Na, sodium; Ca, calcium; Mg, magnesium; Si, silicon; Cr, chromium; Sb, antimony; Sn, tin; K, potassium; ROS, reactive oxygen species; PAHs, polyaromatic hydrocarbons; TSNAs, tobacco-specific nitrosamines; NNN, N-Nitrosonornicotine
